# The effect of Tai Chi on the quality of life in the elderly patients recovering from coronavirus disease 2019: A protocol for systematic review and meta-analysis

**DOI:** 10.1097/MD.0000000000023509

**Published:** 2020-12-04

**Authors:** Ziyu Luo, Ying Chen, Lina Wang, Wenxin Chi, Xiaoxuan Cheng, Xiangyu Zhu

**Affiliations:** a School of Acupuncture and Moxibustion and Tuina; b Dongzhimen Hospital, Beijing University of Chinese Medicine; c Laboratory of Statistics and Measurement, Beijing Sport University, Beijing University of Chinese Medicine, Beijing, China.

**Keywords:** coronavirus disease 2019, meta-analysis, quality of life, systematic review, Tai Chi

## Abstract

**Background:**

coronavirus disease 2019 (COVID-19) is spreading fast starting late 2019. As their cardiopulmonary and immune functions gradually decline, elderly people are prone to COVID-19. Tai Chi has a positive impact on heart function, blood pressure, lung function, blood circulation, and so on, and it's suitable for the elderly. Quality of life (QoL)can reflect of individuals’ physical and mental health, it can also reflects their ability to participate in society. This systematic review and meta-analysis will summarize the current evidence that Tai Chi improve the QoL in the elderly patients recovering from COVID-19.

**Methods:**

We will search PubMed, EMBASE, MEDLINE, the Cochrane Library, Chinese National Knowledge Infrastructure, Chinese Biomedical Literature Database, Chinese Science and Technology Periodical Database, Wanfang Database, Clinical Trials and Chinese Clinical Trial Registry. The complete process will include study selection, data extraction, risk of bias assessment and meta-analyses. Endnote X9.3 will be used to manage data screening. The statistical analysis will be completed by Stata/SE 15.1 software.

**Results:**

This proposed study will evaluate the effectiveness and safety of Tai Chi for the improvement of QoL in elderly COVID-19 patients during the recovery period.

**Conclusion:**

The conclusion of this study will provide evidence to prove the safety and effectiveness of Tai Chi on elderly COVID-19 patients during the recovery period.

**Ethics and dissemination:**

This protocol will not evaluate individual patient information or infringe patient rights and therefore does not require ethical approval.

**Registration:**

PEROSPERO CRD42020206875

## 1 Introduction

The outbreak of coronavirus disease 2019 (COVID-19) raised major concerns, its rapid and widespread spread has affected more than 200 countries and territories, and World Health Organization declared COVID-19 a pandemic on 11 March 2020.^[1,2]^

Compared with other diseases, COVID-19 has a longer incubation period. It's more prone to mutation and more infectious. It has a wider spread, will cause more serious physical and mental problems.^[3]^ Clinical and epidemiological features of COVID-19 demonstrate that the infection can cause clusters of severe respiratory illness, leading to intensive care unit admission and high mortality.^[4]^ A meta-analysis of survivors of severe acute respiratory syndrome and Middle East Respiratory syndrome coronavirus showed: impaired diffusing capacity for carbon monoxide, reduced exercise capacity, prevalences of post-traumatic stress disorder, depression and anxiety are common symptoms 6 months after discharge.^[5]^ Severe acute respiratory syndrome and Middle East Respiratory syndrome exhibit some similarities to COVID-19, but COVID-19 can cause a wider range of symptoms associated with many body systems, such as the heart, kidneys and nervous system, and may have a greater impact on quality of life (QoL).^[6–8]^ In addition, prolonged hospitalization or bedridden treatment for COVID-19 survivors can lead to a sustained reduction in physical activity, which can lead to increased pain and deterioration of joint function.^[9]^ To sum up, COVID-19 will reduce the body function, reduce the exercise ability and have an unhealthy impact on the psychological health, and thus adversely affect the QoL of patients. Studies have shown that immunocompromised individuals and patients with comorbidities are at a higher risk of COVID-19 infection. Compared with young adults, the elderly population has lower cardiopulmonary function and immunity, poor ability to resist virus infection, and a large number of elderly people suffer from complications, thus elderly people are prone to COVID-19.^[10,11]^

So far, more than 20 million people have been confirmed cases of COVID-19, including 911,877 deaths.^[12]^ It is predicted that the disease will reach over 2.4 billion people, with 10.4 million deaths and approximately 2.3 billion recoveries worldwide.^[13]^ Although it is too early to establish the long-term effects of the infection, medium- to long-term damage is expected.^[14]^ Vast number of patients during the recovery period need safe and effective ways to help them recover.

At present, exercise therapy is widely used to restore the body. Studies have shown that exercise therapy can reduce pain and depressive symptoms in patients with muscle fiber pain, and is beneficial in clinically relevant outcomes in patients with juvenile idiopathic arthritis.^[15,16]^ Tai Chi, one of the traditional Chinese exercise therapies, pay attention to body, breath and mind, “three tones in one” to keep healthy. Clinical practice has proved that Tai Chi can improve the functions of the viscera, regulate the nervous system, enhance the resistance of the human body, improve the coordination of the body.^[17–20]^ Existing research proves that Tai Chi can significantly improve test results for asthma and COPD patients on a 6-minute walk, dyspnea, and forced expiratory volume in 1 second.^[21–26]^ Therefore, Tai Chi has outstanding advantages for the respiratory symptoms of COVID-19. Tai chi can also adjust the psychological and mental state. Preliminary trials have shown Tai Chi benefits for schizophrenia, posttraumatic stress disorder, and might be helpful for anxiety.^[27,28]^ The effect of Tai Chi on improving aerobic capacity is also obvious. It may improve exercise capacity in the short, mid, and long terms.^[21]^ So that Tai Chi can be used for COVID - 19 patients to improve QoL. Besides, Tai Chi is a high safety exercise therapy, which is suitable for the elderly. It's effective for preventing falls in older adults.^[29,30]^

In addition, QoL is the perceptions of individuals of their position in life in the context of the culture and value systems they live in and in relation to their objectives, expectations, standards and concerns. QoL can be a comprehensive reflection of individuals’ physical and mental health, and their ability to participate in society.^[31]^ Many subsequent studies have shown that Tai Chi can improve the QoL after multiple diseases.^[32–34]^ The scales can quantitatively reflect the QoL. There are a variety certified scales used to evaluate the QoL, such as 36-item Short Form Survey (SF-36), Nottingham Health-Profile (NHP) and so on. Therefore, we will investigate the effect of Tai Chi on the QoL of the elderly patients with COVID-19 in recovery period in a systematic review and meta-analysis.

## 2 Methods

### 2.1 Registration

The study protocol has been registered on international prospective register of systematic review (PROSPERO registration number: CRD42018094920). The procedure of this protocol will be conducted according to the Preferred Reporting Item for Systematic Review and Meta-analysis Protocols (PRISMA-P) guidance.^[35]^

### 2.2 Inclusion and exclusion criteria

#### 2.2.1 Type of study

Randomised controlled trials (RCTs) about Tai Chi for COVID-19 in recovery period will be included. Non-RCTs, quasi-RCTs, case series, reviews, animal studies and any study with a sample size of less than ten participants will be excluded.

#### 2.2.2 Type of participant

Elderly COVID-19 patients (over 65 years old) who have been clearly diagnosed and now in recovery period, regardless of sex, age, race or educational and economic status, will be included in the review.

#### 2.2.3 Type of interventions

Interventions can be any type of Tai Chi, such as Yang's tai Chi, Chen's Tai Chi and other types of Tai chi. Multiple control measures will be included, such as blank, placebo, usual or standard care, health education, psychosocial therapy, drug therapy. Any comparisons between a combined therapy of Tai Chi exercises and other interventions with a therapy of solely using other interventions are also included. All the frequencies, durations, and types of Tai Chi exercises will be considered.

#### 2.2.4 Type of outcome measures

Outcome indicators include effectiveness indicators and safety indicators. Effectiveness indicators include primary outcome indicators and secondary outcome indicators. The primary outcome indicators are the scores of QoL using validated tools such as SF-36, NHP, World Health Organization QoL scale (whoqol-1000), the QoL index (QL). The secondary outcome indicators are the disappearance time of main symptoms (including fever, asthenia, cough disappearance rate, and temperature recovery time), serum cytokine levels, disappearance time or rate of accompanying symptoms (such as myalgia, expectoration, stuffiness, runny nose, pharyngalgia, anhelation, chest distress, dyspnea, headache, nausea, vomiting, anorexia, diarrhea) negative COVID-19 results rates on two consecutive occasions (not on the same day), computed tomography image improvement, average hospitalization time, occurrence rate of common type to severe form, clinical cure rate, and mortality. Safety are referred to the incidence of adverse events (bleeding, pain, hematoma, syncope, etc).

### 2.3 Search strategy

The following electronic bibliographic databases will be searched to identify relevant studies: PubMed, EMBASE, MEDLINE, the Cochrane Library, Chinese National Knowledge Infrastructure, Chinese Biomedical Literature Database, Chinese Science and Technology Periodical Database, Wanfang Database, Clinical Trials and Chinese Clinical Trial Registry. A combination of subject words and free text words will be applied in the searches. The references of systematic reviews and literature included will also be checked. The search strategies for selecting the fields of title, abstract or keyword will be adjusted according to different characteristics of databases. The language is limited to Chinese and English. The search terms are shown in Table 1.

**Table 1:** Search strategy of PubMed.

Number	Search items
1	COVID-19^∗^. Mesh.
2	COVID-19. ti, ab
3	COVID19. ti, ab
4	SARS-CoV-2. ti, ab
5	2019-nCoV. ti, ab
6	2019 nCoV. ti, ab
7	2019nCoV. ti, ab
8	2019 novel coronavirus. ti, ab
9	coronavirus disease 2019. ti, ab
10	coronavirus disease-19. ti, ab
11	Wuhan seafood market pneumonia virus. ti, ab
12	Wuhan coronavirus. ti, ab
13	1 or 2–12
14	Tai Ji. Mesh.
15	Tai Ji. ti, ab
16	Tai-ji. ti, ab
17	Tai Chi. ti, ab
18	Chi, Tai. ti, ab
19	Tai Ji Quan. ti, ab
20	Ji Quan, Tai. ti, ab
21	Quan, Tai Ji. ti, ab
22	Taiji. ti, ab
23	Taijiquan. ti, ab
24	T’ai Chi. ti, ab
25	Tai Chi. ti, ab
26	14 or 15-25
27	randomized controlled trial. pt
28	randomized. ti, ab
29	placebo. ti, ab
30	23 or 27 or 28
31	13 and 26 and 29

∗ COVID-19 = coronavirus disease 2019, SARS = severe acute respiratory syndrome.

### 2.4 Study selection

**Figure 1:**
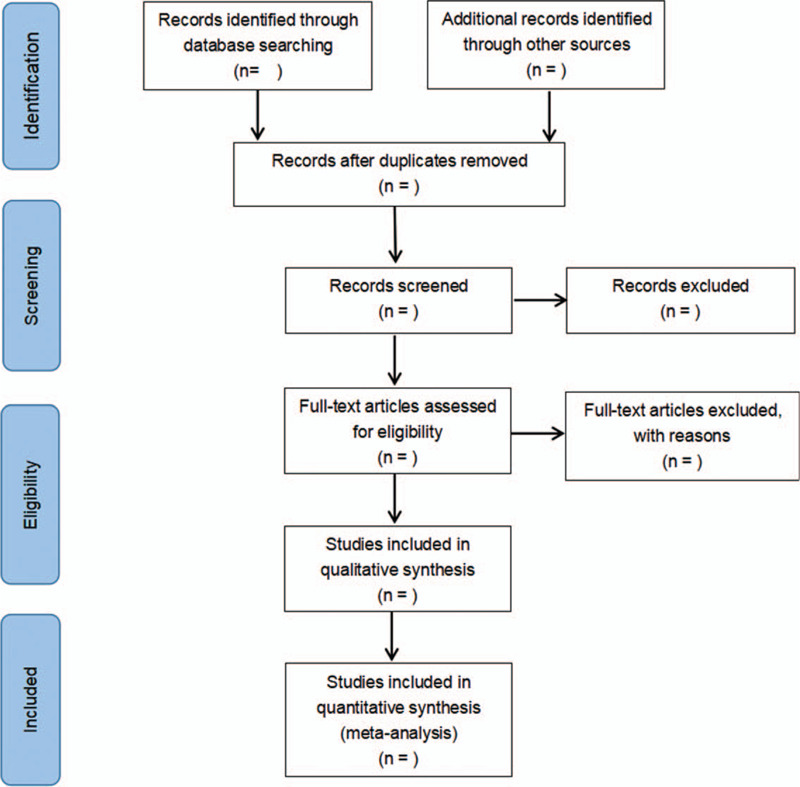
The PRISMA flow chart presenting details of the selection process.

The literature will be retrieved according to the retrieval strategy, then imported them into the literature management software. Endnote version 9.3 (The Thomson Corporation Corp, Stanford, CT) will be used to manage data screening. The research on duplicate titles was deleted, and obviously irrelevant literature was excluded by reading titles and abstracts. The above steps were performed independently by 2 researchers. Any disagreements will be resolved by discussion with a third researchers. The researchers will record all studies that do not meet the inclusion criteria and provide the rationale for their exclusion. Details of the selection process will be presented in the PRISMA flow chart. (Fig. 1)

### 2.5 Data extraction

Extract data from selected studies, which include general information, reference (name of the leading author and year of publication, and study design), participant characteristic, intervention, methods, control, training frequency and length, outcomes measured, results, adverse reactions. The above steps were performed independently by two researchers. Any disagreements will be resolved by discussion with a third researchers. If required information is not reported, we will try our best to contact the corresponding authors of the studies through email to deal with missing data. And the study will be further excluded without adequate information.

### 2.6 Risk of bias assessment

Two authors will assess methodological quality of included studies separately by the Cochrane collaboration's risk of bias tool.

We will consider the following:

(1) random sequence generation (selection bias)
(2) allocation concealment (selection bias)
(3) blinding of participants and personnel (performance bias)
(4) blinding of outcome assessment (detection bias)
(5) incomplete outcome data (attrition bias)
(6) selective reporting (reporting bias)
(7) other sources of bias (other bias).

The bias risk in each aspect will be assessed and divided into 3 levels: low risk, high risk, and unclear risk.

### 2.7 Statistical analysis

#### 2.7.1 Strategy for data synthesis

Stata/SE version 15.1 (STATA Crop., College Station, TX) will be used to conduct this meta-analysis. The groups included in synthesis must meet our inclusion criteria, using a recognised QoL scale. The data results will be calculated as mean difference or standardized mean difference with corresponding 95% confidence intervals. Heterogeneity will be assessed using the *Q* test (with *P* < .1 considered to represent significant statistical heterogeneity), and the *I*^2^ statistic (with *I*^2^ > 50% considered to be indicative of substantial heterogeneity). If necessary, meta-regression, subgroup and sensitivity analyses will also be performed to analyze the source of any heterogeneity. Data synthesis calculated using a random-effects or fixed-effects mode. We will clearly describe which studies were included and how they have been synthesised as described. We will be transparent about the metric being used.

#### 2.7.2 Analysis of subgroups or subsets

Subgroup analysis will be performed to explain heterogeneity if necessary, such as different duration of Tai Chi intervention, the inconsistency of scoring systems used to evaluate QoL and different Interventions.

#### 2.7.3 Sensitivity analysis

Different levels of the methodological quality of trails may tend to affect the overall effects. If the *Q* test and the *I*^2^ statistic show significant statistical heterogeneity, sensitivity analyses we will conduct sensitivity analysis. Sensitivity analysis is conducted by excluding studies one by one, so that we can determine the source of heterogeneity.

#### 2.7.4 Publication bias

The publication bias will be evaluated by funnel plots by determining whether there are 10 or more studies with the same outcome. In the case of asymmetric funnel plot, subgroup analysis or sensitivity analysis will be performed to investigate possible causes.

#### 2.7.5 Quality of evidence

We will use the Grading of Recommendations Assessment, Development, and Evaluation guidelines for the assessment of the strength of evidence for each outcome. The result will be categorised as high, moderate, low, and very low certainty of evidence.

### 2.8 Ethics and dissemination

This systematic review will not require ethical approval because there are no data used in our study that are linked to individual patient data.

## 3 Results

This proposed study will evaluate the effectiveness and safety of Tai Chi for the improvement of QoL in elderly COVID-19 patients during the recovery period.

## 4 Discussion

As to 12 September 2020, there have been 28,329,790 confirmed cases of COVID-19, including 911,877 deaths, reported to World Health Organization, from more than 200 countries and territories.^[12]^ While actively treating patients to reduce mortality and developing vaccines to reduce infection rates, should also pay attention to improve the QoL of survivors. Exercise therapy is an active self-healing method, which benefits associated with improved overall physical and mental health, and physical functioning. It may be associated with minimal side effects as compared to drug and surgical interventions.^[36]^ Tai Chi is a traditional physical and mental training. The movements of Tai Chi are lively, continuous and oscillating, which accords with the psychological and physiological characteristics of the elderly.^[37]^ Studies have found that Tai Chi has many positive effects on the health of the elderly, and it is a very suitable exercise for the elderly.^[38,39]^ In the fight against virus infection, it is particularly important to improve the activity of autoimmunity, reduce the body's susceptibility to infectious diseases and strengthen its resistance. Tai Chi exercise can enhance the secretion of erythropoietin and leukocyte stimulating factor in the body, leading to physiological adaptation changes in the blood system, so as to enhance the blood function, especially the immune function. Therefore, Tai Chi has its outstanding advantages in the face of COVID-19.

At present, there are no systematic review of the effects of Tai Chi on QoL in convalescent elderly patients with COVID-19. It is hoped that this meta-analysis can provide convincing scientific basis and guide clinical practice. Nonetheless, the lack of sufficient RCTs may be a limitation for this meta- analysis.

## 5 Conclusion

The conclusion of this study will provide evidence to prove the safety and effectiveness of Tai Chi on elderly COVID-19 patients during the recovery period.

## Author contributions

ZYL participated in the design of the study, literatures collection and analysis; YC participated in data collection; LNLparticipated in Literature data extraction and quality assessment; XYZ, WXC participated in the design of the study and contributed to literatures collection; XXC participated in the design of the study; All authors contributed to the manuscript writing. All authors have read and approved the final version of the manuscript, and agree with the order of presentation of the authors.

**Conceptualization:** Xiangyu Zhu, Wenxin Chi.

**Data curation:** Ying Chen, Lina Wang.

**Formal analysis:** Xiangyu Zhu, Lina Wang.

**Funding acquisition:** Xiangyu Zhu.

**Investigation:** Ziyu Luo, Wenxin Chi, Xiaoxuan Cheng.

**Methodology:** Ziyu Luo, Lina Wang.

**Writing – original draft:** Ziyu Luo.

## Correction

When originally published Moxibustion and Tuina, Beijing University of Chinese Medicine was incorrectly included in affiliation c. This has since been corrected.

## Abbreviations

Abbreviations: COVID-19 = coronavirus disease 2019, QoL = quality of life.
